# Antidepressants, Cognitive Decline and Dementia: A Meta-Analysis with Insights into the Brain–GutAxis

**DOI:** 10.1192/bjo.2026.11246

**Published:** 2026-06-30

**Authors:** Baidyanath Ghosh Dastidar, Prathama Guha, Ashik Zaman

**Affiliations:** 1 Kolkata, Kolkata, India; 2 Calcutta National Medical College, Kolkata, India

## Abstract

**Aims:**

Depression is a recognised risk factor for cognitive decline and dementia, yet the long-term neurocognitive effects of antidepressant treatment remain uncertain. This study aimed to quantify the association between antidepressant use and risk of cognitive decline and dementia, and to explore potential mechanistic links through the brain–gut axis using emerging microbiome evidence.

**Methods:**

We conducted a PRISMA-compliant meta-analysis prospectively registered with PROSPERO (CRD420251012092). The protocol development, literature search, data extraction and verification were completed over a six-month period. PubMed, EMBASE, PsycINFO and the Cochrane Library were searched from inception to March 2025 for randomised controlled trials and longitudinal cohort studies examining antidepressant exposure and cognitive decline or dementia outcomes. Two reviewers independently screened studies and extracted data using a pre-specified framework. All extracted data were manually cross-checked against the original full-text publications to ensure accuracy and consistency. Random-effects meta-analyses were performed to estimate pooled effects, with subgroup, sensitivity and meta-regression analyses conducted to explore age, treatment duration and antidepressant class. Randomised microbiome studies were narratively synthesised to examine potential brain–gut mechanisms.

**Results:**

Seventy-eight studies met inclusion criteria, comprising 102,584 participants (22 randomised controlled trials and 56 longitudinal cohort studies). Antidepressant use was associated with a significantly reduced risk of dementia (odds ratio 0.85, 95% CI 0.78–0.92) and slower cognitive decline (standardised mean difference 0.28, 95% CI 0.15–0.41). Subgroup analyses demonstrated stronger protective effects among individuals aged under 65 years and in those receiving antidepressant treatment for more than three years. Selective serotonin reuptake inhibitors showed the most consistent association with reduced dementia risk and cognitive decline compared with other antidepressant classes. Heterogeneity was moderate to substantial and consistent with differences in study design, follow-up duration and outcome definitions. Narrative synthesis of five microbiome randomised trials showed that antidepressant-associated increases in Bifidobacterium and reductions in inflammatory markers correlated with improvements in cognitive performance, supporting a potential gut–brain mechanism underlying observed neuroprotective effects.

**Conclusion:**

Antidepressant use is associated with a modest but statistically significant reduction in dementia risk and slower cognitive decline, particularly with long-term treatment and selective serotonin reuptake inhibitors. Integration of emerging microbiome evidence suggests that anti-inflammatory and gut-mediated mechanisms may contribute to these effects. While causality cannot be inferred, these findings support further prospective and mechanistic studies to clarify the role of antidepressants in cognitive ageing and dementia prevention.

